# Solving Stochastic Reaction Networks with Maximum Entropy Lagrange Multipliers

**DOI:** 10.3390/e20090700

**Published:** 2018-09-12

**Authors:** Michail Vlysidis, Yiannis N. Kaznessis

**Affiliations:** Department of Chemical Engineering and Materials Science, University of Minnesota, Minneapolis, MN 55455, USA

**Keywords:** stochastic chemical reactions, probability distributions, maximum entropy, lagrange multipliers

## Abstract

The time evolution of stochastic reaction networks can be modeled with the chemical master equation of the probability distribution. Alternatively, the numerical problem can be reformulated in terms of probability moment equations. Herein we present a new alternative method for numerically solving the time evolution of stochastic reaction networks. Based on the assumption that the entropy of the reaction network is maximum, Lagrange multipliers are introduced. The proposed method derives equations that model the time derivatives of these Lagrange multipliers. We present detailed steps to transform moment equations to Lagrange multiplier equations. In order to demonstrate the method, we present examples of non-linear stochastic reaction networks of varying degrees of complexity, including multistable and oscillatory systems. We find that the new approach is as accurate and significantly more efficient than Gillespie’s original exact algorithm for systems with small number of interacting species. This work is a step towards solving stochastic reaction networks accurately and efficiently.

## 1. Introduction

The traditional approach to describe chemically reacting systems involves the use of deterministic rate laws. This macroscopic, continuous-deterministic modeling formalism is appropriate at the thermodynamic limit, when the volume of the system and the numbers of molecules of reactants and products all tend to very large values [1]. However, this approach fails to describe molecular populations that are small, finite and countable [2]. Such populations must be described by variables that can only change stochastically and by discrete amounts [3,4,5,6,7,8].

Markov chain models can be used to describe chemical reactions away from the thermodynamic limit [9]. When the system evolves stochastically, the all-encompassing chemical master equation (CME) can model the probability distribution of the system being at a particular state at time *t* [3,4].

The historical difficulties in solving the CME are well documented in the literature [5,9,10,11]. The CME is not a single equation but an infinite set of coupled equations. As a result the CME is analytically unsolvable for the majority of systems, with only few exceptions [12,13]. An established way to sample the master probability distribution is to use Gillespie’s Stochastic Simulation Algorithm (SSA) [6]. Despite the accuracy of SSA, the method is a kinetic Monte Carlo algorithm and hence computationally expensive, especially for systems with large numbers of molecules, or with reaction kinetics that span multiple time scales [14,15].

A proposed alternative to solve the CME is by calculating the probability moments [3]. Moments are expected values, e.g., the mean and the variance of the number of molecules [5,16,17,18]. This approach is based on the concept that any probability distribution can be completely described by its moments [19]. The starting point of moment equations is to rewrite the CME in terms of moments describing the master probability distribution [18,20]. Instead of a master equation that governs the probability distribution in time, one can then write a set of ordinary differential equations evolving the moments of the probability distribution [15,21]. Efficient algorithms have been developed to generate these moment equations for arbitrary networks [10,15,17,18,21]. Yet, because the dynamics of lower-order moments depend on the higher ones for non-linear reaction networks, the system of ODEs needs to be closed or somehow truncated in order to be solved [18,22].

A common approach to tackle the moment equation closure challenge is to a priori assume a specific form of the species behavior. By assuming a molecular component’s behavior one can relate the higher order moments to the lower order ones [19,23,24]. An important subset of the literature approaches the issue by assuming a specific form of the system’s probability distribution, such as normal, Poison, lognormal, Gamma etc. [10,21,24,25,26]. In cases when the molecular components behave in a known fashion, such distribution assumptions may produce reasonably accurate results. However, in most systems, there is a lack of knowledge of the species time evolution and thus such assumptions may break down or become impractical.

Recent advances in probability moment equation closure schemes have made it possible to efficiently calculate the stationary probability distribution [22]. These methods may quite accurately calculate the stationary behavior, however, they still face significant numerical challenges calculating the time evolution of the moments [15,19,27].

To circumvent the challenges of solving moment equations in time, we propose the use of Lagrange multipliers [22,28,29]. The connection between moments and Lagrange multipliers relies on the maximum entropy principle, which states the system attains a probability distribution that maximizes its entropy [20,22,28,30,31].

By Shannon’s definition entropy is given by $S=-\sum_{X}P\left(X\right)logP\left(X\right)$ [30], where X is the number of molecules for each component and *P* the probability function. Using this definition and assuming that *S* is maximum, the system of moment equations can be transformed into a system of equations that depend only on the Lagrange multipliers [20,22]. We refer to the new system as “Lagrange multiplier equations (LMEs)”. The LMEs set, unlike moment equations [3,18], is a closed system, i.e., it has the same number of unknowns with equations. Thus, an intial value problem numerical technique, like Runge-Kutta [32], can be used to solve the system in time.

Herein, we unpack in full mathematical detail the derivation of a general system of LMEs in the next section. We then apply the LMEs method to multiple examples of non-linear reaction networks, including the bistable Schlögl model [33], the multistable Wilhelm model [34], the oscillatory Brusselator system [35,36] and the viral infection model [37,38]. After presenting a numerical procedure for the implementation of the method, we present results comparing the accuracy of LMEs solutions to SSA simulations. We conclude that the significance of this new method is that LMEs can solve reaction networks as accurate as SSA with significantly less computational cost. SSA has been used widely in the past two decades to capture the stochastic nature of chemical and biological systems away from the thermodynamic limit. LMEs may offer a potentially more efficient alternative to SSA, enabling practitioners to model stochastic reacting networks.

## 2. Materials and Methods/Theory

### 2.1. Lagrange Multiplier Equations

#### 2.1.1. Connect Moments to Lagrange Multipliers

The general form of the moment equations for any arbitrary chemical reaction network is:

(1) $$\frac{\partial \mu }{\partial t}=A\mu +A^{\prime }\mu^{\prime }+\mu_{c}$$

where μ is the lower-order moment vector (not including the zeroth-order moment, which is always equal to 1), $\mu^{\prime }$ is the higher-order moment vector, $\mu_{c}$ is a vector of constants and *t* represents time. *A* and $A^{\prime }$ are constant matrices that represent the linear and non-linear components of the network, respectively. In most cases, the vectors μ and $\mu^{\prime }$ have different dimensions [20]. For non-linear reaction networks, the $\mu^{\prime }$ vector is nonempty.

For a chemical reaction network with *N* reactants and products, the moment $\mu_{i}$ is connected to the probability function *P* through the following expression:

(2) $$\mu_{i}=\sum_{\Omega }f_{\mu_{i}}\left(X\right)P\left(X\right)$$

where $f_{\mu_{i}}$ is the functional form of the *i*th moment $\mu_{i}$. For example, for a one component system the functional form of the 3rd polynomial moment is $f_{\mu_{3}}=Y^{3}$, where *Y* is the system’s component. The number of molecules of each component is contained in the matrix $X=(X_{1}\ldots X_{N})$ and Ω corresponds to the *N*-dimensional state space for all the possible values of $\left(X_{1}\ldots X_{N}\right)$. The differences between X and Ω becomes clear if one compares Equation (2) and the definition of entropy ($S=-\sum_{X}P\left(X\right)logP\left(X\right)$).

One can connect the probability distribution to Lagrange multipliers λ by maximizing the entropy [22]:

(3) $$P\left(X\right)=\exp\left[-\sum_{j=0}^{M}\lambda_{j}f_{\mu_{j}}\left(X\right)\right]$$

where $\lambda_{j}$ is the *j*th Lagrange multiplier and *M* is the number of lower-order moments and also the size of vector μ. Thus, moments can be related to Lagrange multipliers by combing Equations (2) and (3):

(4) $$\mu_{i}=\sum_{\Omega }\left\{f_{\mu_{i}}\left(X\right)\exp\left[-\sum_{j=0}^{M}\lambda_{j}f_{\mu_{j}}\left(X\right)\right]\right\}$$

#### 2.1.2. Time Derivatives

In Equation (3), only the Lagrange multipliers depend on time; the state space and the functional form of the moments are time-independent. Hence, the time derivative of the probability distribution is:

(5) $$\begin{array}{cc} \frac{\partial P(X)}{\partial t} & =\frac{\partial \exp\left[-\sum_{j=0}^{M}\lambda_{j}f_{\mu_{j}}\left(X\right)\right]}{\partial t}\\ & =-\exp\left[-\sum_{j=0}^{M}\lambda_{j}f_{\mu_{j}}\left(X\right)\right]\left[\sum_{j=0}^{M}\frac{\partial \lambda_{j}}{\partial t}f_{\mu_{j}}\left(X\right)\right] \end{array}$$

This equation and the definition of moments (Equation (2)) connect the time derivative of the moments to the time derivative of the Lagrange multipliers:

(6) $$\begin{array}{cc} \frac{\partial \mu_{i}}{\partial t} & =\frac{\partial \sum_{\Omega }f_{\mu_{i}}\left(X\right)P\left(X\right)}{\partial t}\\ & =\sum_{\Omega }f_{\mu_{i}}\left(X\right)\frac{\partial P(X)}{\partial t}\\ & =-\sum_{\Omega }\left\{f_{\mu_{i}}\left(X\right)\exp\left[-\sum_{j=0}^{M}\lambda_{j}f_{\mu_{j}}\left(X\right)\right]\left[\sum_{j=0}^{M}\frac{\partial \lambda_{j}}{\partial t}f_{\mu_{j}}\left(X\right)\right]\right\}\\ & =-\sum_{j=0}^{M}\left(\sum_{\Omega }\left\{f_{\mu_{i}}f_{\mu_{j}}\left(X\right)\exp\left[-\sum_{j=0}^{M}\lambda_{j}f_{\mu_{j}}\left(X\right)\right]\right\}\frac{\partial \lambda_{j}}{\partial t}\right) \end{array}$$

Thus, the moments’ time derivative is transformed to:

(7) $$\frac{\partial \mu_{i}}{\partial t}=-\sum_{j=0}^{M}\left(\mu_{i,j}\frac{\partial \lambda_{j}}{\partial t}\right)$$

where $\mu_{i,j}$ is the combined moment of $f_{\mu_{i}}$ and $f_{\mu_{j}}$, given by

(8) $$\mu_{i,j}=\sum_{\Omega }\left\{f_{\mu_{i}}f_{\mu_{j}}\left(X\right)\exp\left[-\sum_{j=0}^{M}\lambda_{j}f_{\mu_{j}}\left(X\right)\right]\right\}$$

The sum of the probabilities across the whole state space is always 1 by definition. As a result, the zeroth-order moment is 1 ($\mu_{0}=1$) and its time derivative is zero ($\frac{\partial \mu_{0}}{\partial t}=1$). This results in the zeroth Lagrange multiplier $\lambda_{0}$ to be dependent on the rest of Lagrange multipliers

(9) $$\begin{array}{cc} 0 & =\frac{\partial \mu_{0}}{\partial t}\\ & =-\sum_{j=0}^{M}\left(\mu_{j}\frac{\partial \lambda_{j}}{\partial t}\right)\\ & =-\mu_{0}\frac{\partial \lambda_{0}}{\partial t}-\sum_{j=1}^{M}\left(\mu_{j}\frac{\partial \lambda_{j}}{\partial t}\right)\\ & =-\frac{\partial \lambda_{0}}{\partial t}-\sum_{j=1}^{M}\left(\mu_{j}\frac{\partial \lambda_{j}}{\partial t}\right) \end{array}$$

Hence, the time derivative of the zeroth Lagrange multiplier depends on the rest of the time derivatives as follows:

(10) $$\frac{\partial \lambda_{0}}{\partial t}=-\sum_{j=1}^{M}\left(\mu_{j}\frac{\partial \lambda_{j}}{\partial t}\right)$$

Based on this relation, Equation (7) can be modified as:

(11) $$\begin{array}{cc} \frac{\partial \mu_{i}}{\partial t} & =-\sum_{j=0}^{M}\left(\mu_{i,j}\frac{\partial \lambda_{j}}{\partial t}\right)\\ & =-\mu_{i}\frac{\partial \lambda_{0}}{\partial t}-\sum_{j=1}^{M}\left(\mu_{i,j}\frac{\partial \lambda_{j}}{\partial t}\right)\\ & =\mu_{i}\sum_{j=1}^{M}\left(\mu_{j}\frac{\partial \lambda_{j}}{\partial t}\right)-\sum_{j=1}^{M}\left(\mu_{i,j}\frac{\partial \lambda_{j}}{\partial t}\right)\\ & =\sum_{j=1}^{M}\left(-\mu_{i,j}+\mu_{i}\mu_{j}\right)\frac{\partial \lambda_{j}}{\partial t} \end{array}$$

We have previously proved [39] that:

(12) $$-\mu_{i,j}+\mu_{i}\mu_{j}=\frac{\partial \mu_{i}}{\partial \lambda_{j}}$$

Hence, the time derivative of one moment is given by (Equations (11) and (12)):

(13) $$\frac{\partial \mu_{i}}{\partial t}=\sum_{j=1}^{M}\frac{\partial \mu_{i}}{\partial \lambda_{j}}\frac{\partial \lambda_{j}}{\partial t}$$

or in matrix form:

(14) $$\frac{\partial \mu }{\partial t}=\frac{\partial \mu }{\partial \lambda }\frac{\partial \lambda }{\partial t}=J\frac{\partial \lambda }{\partial t}$$

*J* represents the Jacobian matrix of the system. Equation (14) also represents the chain rule of differentiation. There is now a way to connect the moment equations to equations that involve the Lagrange multipliers. The Lagrange multiplier equations can be derived by Equation (14) and the moment equations (Equation (1)):

(15) $$\frac{\partial \lambda }{\partial t}=J^{-1}\frac{\partial \mu }{\partial t}=J{\left(\lambda \right)}^{-1}\left[A\mu (\lambda )+A^{\prime }\mu^{\prime }\left(\lambda \right)+\mu_{c}\right]$$

where $J^{-1}$ is the inverse of the Jacobian matrix. In the Appendix A, we discuss a computationally efficient way to calculate it. Even though the above equation also includes the moments of the probability distribution, it only depends on the Lagrange multipliers since the moments are directly correlated with Lagrange multipliers (Equation (4)).

All the Lagrange multipliers can be calculated through Equation (15) except the zeroth-order one $\lambda_{0}$. As discussed earlier, $\lambda_{0}$ depends on the rest of the Lagrange multipliers through the relation [39]:

(16) $$\lambda_{0}=\log\left\{\sum_{\Omega }\left[\exp\left(-\sum_{j=1}^{M}\lambda_{j}f_{\mu_{j}}\left(X\right)\right)\right]\right\}$$

We refer to Equation (15) as the “Lagrange multiplier equations (LMEs)”. To our knowledge, this is the first time that LMEs are being discussed and derived in the literature. Through this step, the problem of calculating the probability for each point of the state space of stochastic networks has been transformed into a problem of calculating a finite set of Lagrange multipliers. The system is closed, it has the same number of equations and unknowns, and it is an initial value problem. An iterative numerical method can be used to solve this system. Appendix B outlines a proposed numerical approach to solve LMEs based on the Dormand-Prince Runge-Kutta RK5(4)7M method [32].

### 2.2. Initial Value from Moments to Lagrange Multipliers

The LMEs problem is an initial value problem; the values of the Lagrange multipliers for the initial state are required. However, Lagrange multipliers are numerical variables with no physical meaning and as a result an initial value is unknown. What is known is either the initial probability distribution or the values of its moments. Hence, a way to calculate Lagrange multipliers from moments is required. It should be noted that there is not a universally acceptable way to back calculate probability distributions from moments and the problem is not trivial [40].

Let’s assume that the moments, $\mu \left(t=0\right)$ of the initial distribution, $P\left(t=0\right)$ are known. If only the initial distribution is known, its moments can be calculated from Equation (2). The moments are related to the Lagrange multipliers by Equation (4). Hence:

(17) $$\begin{array}{cc} \mu \left(t=0\right) & =\left[\begin{array}{c} \mu_{1}\left(t=0\right)\\ \mu_{2}\left(t=0\right)\\ \vdots \end{array}\right]\\ & =\left[\begin{array}{c} \sum_{\Omega }\left\{f_{\mu_{1}\left(t=0\right)}\left(X\right)\exp\left[-\sum_{j=0}^{M}\lambda_{j}\left(t=0\right)f_{\mu_{j}\left(t=0\right)\left(t=0\right)}\left(X\right)\right]\right\}\\ \sum_{\Omega }\left\{f_{\mu_{2}\left(t=0\right)}\left(X\right)\exp\left[-\sum_{j=0}^{M}\lambda_{j}\left(t=0\right)f_{\mu_{j}\left(t=0\right)\left(t=0\right)}\left(X\right)\right]\right\}\\ \vdots \end{array}\right]\\ & =G\left[\lambda \left(t=0\right)\right] \end{array}$$

***G*** denotes the functional form of the matrix with dependences of the Lagrange multipliers. To calculate the Lagrange multipliers at the initial time from the moments, one has to solve a problem of the form: $G\left(\lambda \right)-\mu =0$. This is an infinite set and in order to be numerically solvable the user should decide the necessary number of lower-order moments (i.e., to specify the closure order [22]). When closure order is specified, the system has the same number of equations and unknowns (the Lagrange multipliers) and a root-finding method such as Newton-Rapshon can be used. The residual of the method is $R=G\left(\lambda \right)-\mu$ and the Jacobian matrix is $J_{i,j}=\frac{\partial F_{i}}{\partial \lambda_{j}}=\frac{\partial \mu_{i}}{\partial \lambda_{j}}=-\mu_{i,j}+\mu_{i}\mu_{j}$ [39]. With this approach all the Lagrange multipliers can be calculated other than the zeroth one. The zeroth one can be calculated from Equation (16).

The knowledge of moments is used here to calculate the Lagrange multipliers, which then can be used to calculate the probability distribution based on Equation (3). This approach allows to calculate probability distributions from its moments. The novelty of the method is the connection of the probability distribution with Lagrange multipliers by maximizing the entropy of the system.

## 3. Results

To demonstrate the proposed Lagrange multiplier equations (LMEs) approach, we employ four different example networks: the bistable Schlögl model [33], the multistable Wilhelm’s system [34], the oscillatory Brusselator [35,36] and the viral infection [37,38]. The reaction networks and kinetic constants for each model are reported in Table 1. To assess the accuracy and computational efficiency of this method, the results are compared to SSA results using the same initial condition and kinetic constants.

For all the systems the initial condition is a distribution generated with SSA, away from the steady state. The first and second-order factorial moments [17] of each initial distribution can be found in Table 1. Using the algorithm in Section 2.2, the initial distribution was transformed into an initial set of Lagrange multipliers. Based on the initial Lagrange multipliers and the Prince-Dormant algorithm outlined in Appendix B, the Lagrange multipliers for every time point were calculated until steady state was reached. Equations (3) and (4) were then used to calculate the probability distribution and factorial moments for each time point based on the calculated Lagrange multipliers.

All the LMEs results obtained with RK5(4)M are compared with SSA results on Table 2, in order to draw conclusions about the accuracy and the time efficiency of the method. The solution for each time point was compared to the one obtained from SSA simulations with 500,000 trajectories. The average difference among all time points between the two methods can be found in Table 2. Results for both the probability distribution and the first two orders of factorial moments are presented. For the system with more than one components (i.e., Wilhelm’s, Brusselator and viral infection models), the moments of the same order are averaged. The difference between LMEs results and the SSA probability distribution is calculated with the Kullbalck-Leiber divergence [41], whereas the second norm is used to calculate the errors in the moments.

### 3.1. Multistable Systems: The Schlögl and Wilhelm Models

The Schlögl model is one of the most commonly used theoretical bistable systems and is the simplest single-component system that can exhibit bistability [9,34]. Biological systems exhibiting bistability, both natural and synthetic, have gained an increasing interest recently [42,43,44], and the Schlögl model is a simple prototypical bistable model.

**Figure 1:**
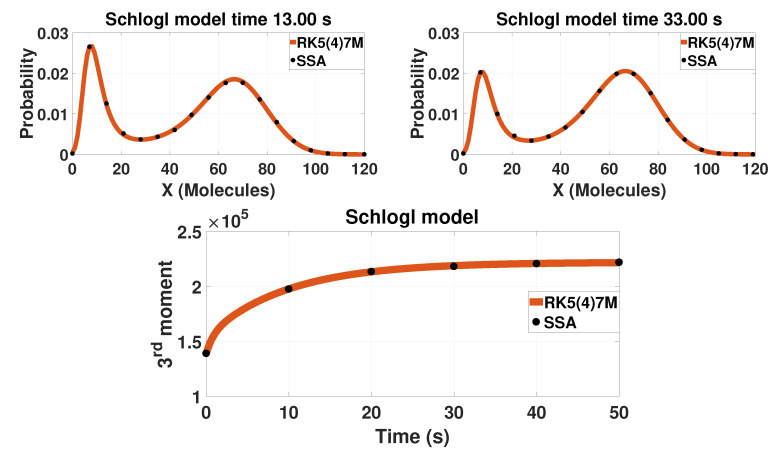
The probability distribution and the third-order moment of the bistable Schlögl model. The first row presents the probability distribution of the single component for two different time points (13 s after the initial condition the left and 33 s the right one). The second row presents the time evolution of the third moment. The solid lines are solutions calculated based on LMEs with tenth-order closure (M=10). The closure order is chosen based on the findings of [29]. The dots represent results from 500,000 SSA trajectories.

For different kinetic constants, the model has either a unimodal or a bimodal distribution. The kinetic constants selected in Table 1 result in a bimodal network. Results for the bistable model are reported on Figure 1. The probability distribution of the model is a bimodal distribution that changes form with time.

**Figure 2:**
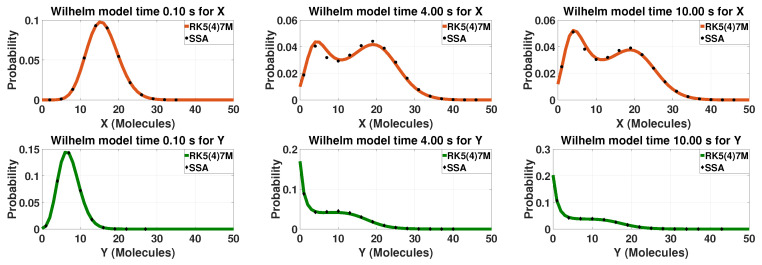
The probability distributions of the multistable Wilhelm’s model. The first row presents results for component *X* and the second row for component *Y*. The first column presents the time close to the initial condition (0.1 s), the second a mid-time point (4 s) and the last column a time point close to steady-state (10 s). The solid lines are solutions calculated based on LMEs and the dots/diamonds SSA solutions. The LMEs are calculated with up to sixth-order moments (M=27). The closure order is chosen based on the findings of [39,45]. For SSA, 500,000 trajectories were used.

Another example of a theoretical system that can exhibit bistability is Wilhelm’s network. The network is a non-linear two-component model and it was created by Wilhelm as the smallest known bistable chemical reaction system [34]. Depending on the kinetic constants, both components can exhibit bistability simultaneously which results in a multistable system overall. This is the case for the kinetic constants reported in Table 1. The probability distribution for different time points for the two component of the network can be found in Figure 2. The system starts with a unimodal distribution for both components. As the time progresses, both components have a different bimodal distribution resulting in a mutlistable network. Close to the steady state, both components still have bimodal distributions but different than before.

For these two example systems, the LMEs solutions are as accurate as the SSA results (Table 2). All the probability and moments errors are lower than 1%. The accuracy of the LMEs approach does not seem to be affected by the number of stability points of the system. There is also no significant difference between the first and second-order moment errors.

### 3.2. Oscillatory System: The Brusselator Model

Oscillatory behaviors are particularly challenging to capture with stochastic models [46]. We have reported earlier how methods that can capture multistable behaviors fail to appropriately capture oscillatory ones [39].

**Figure 3:**
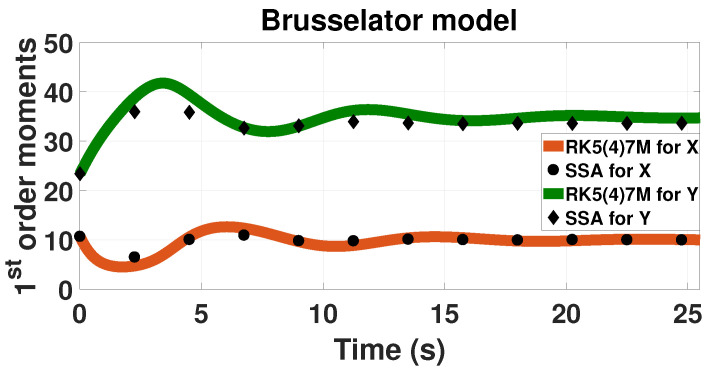
Time evolution of the first-order probability moments for the oscillatory Brusselator model. The solid-line upper green line presents the moment for *Y* component calculated based on LMEs. The solid-line lower orange line represents the moment for *X*. SSA results are also included from 500,000 trajectories; dots represent component *X* and diamonds component *Y*. The results are calculated with up to fourth-order moments (M=14).

We used the LMEs to investigated the Brusselator as a simple example of a theoretical oscillatory network [36]. We study the network in its oscillatory region as shown in Figure 3. Both network’s first-order moments oscillate with respect to time.

As observed, the LMEs can capture the damped oscillatory behavior of the Brusselator. However, the method exhibits limited accuracy for solving this model (Table 2). The solution by LMEs of the Brusselator network has an increased error in all categories compared to the other networks. The error in both probability and moments reaches up to 10%. In this case, unlike in the other networks, there is a significant increase in the error of the second-order moments compared to the first-order ones.

The difference in the accuracy can be associated with the order of closure that is used to generate the LMEs (in this case 4 or M=14, Figure 3). The accuracy of the Lagrange multipliers approach depends on the number of lower-order moments, *M* [29]. The higher the value of *M*, the more accurate results the method can produce. After, a certain value of *M*, the improvement in accuracy is insignificant. For more information on how the lower-order moments affects probability distributions the reader is directed to the literature [29]. It has been reported that the Brusselator requires more than fourth-order moments for accurate results [46].

A low order is used for this system due to the numerical difficulties of the algorithm that generates the initial condition (Section 2.2). In the LMEs approach, the closure order is specified through the initial condition. This is the number of Lagrange multipliers with known values at the initial time. For the rest of the time points, the number of Lagrange multipliers is the same as the initial time. For this system, the initial probability distribution was created with SSA and then, based on the algorithm described in Section 2.2, it was translated into the initial Lagrange multipliers. However, the algorithm in Section 2.2 faced significant numerical issues for moment orders higher than 4 and we were not able to generate initial conditions for higher lower-order moments. Thus, we did not provide the LMEs approach with the necessary number of lower-order moments that produce accurate results. The high error was generated by the algorithm that calculates the initial condition and not the Runge-Kutta algorithm of the LMEs. The Brusselator results are not ideal; however, given the difficulties of current methods to solve oscillatory systems, this is a step in the right direction.

### 3.3. Multicomponent System: The Viral Infection Model

**Figure 4:**
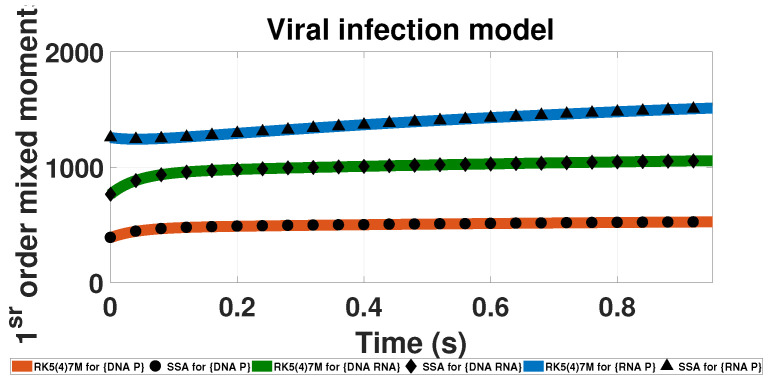
Time evolution of the first-order mixed probability moments for the Viral infection model. The solid-lines present results calculated based on LMEs and the doted points present SSA results. The results are calculated with up to second-order moment closure (M=9) and 500,000 SSA trajectories. The upper blue line with triangles present the {DNAProtein} mixed moment, the mid green line with diamonds corresponds to the {DNARNA} mixed moment and the lower orange line with dots the {RNAProtein} mixed moment.

The LMEs approach is only limited to theoretical systems with complex behavior but can also be applied to natural and synthetic biological networks. One example is the viral infection model. The model was first created by Haseltine [37] as a general model of a cell infection by a virus. The model was then revised and simplified by Goutsias [38]. The network in Table 1 reflects the revised version of Goutsias. Results for the mixed first-order moments (moments than depend on more than one component) are reported in Figure 4. The LMEs approach accurately captures the different dynamics of all components and their combinations throughout the entire simulated time (Table 2).

### 3.4. Computational Cost of LMEs

Aside from the accuracy of the approach, Table 2 also includes the computational time that is required for RK5(4)M to solve the LMEs. In all the systems, the LMEs approach is significantly faster than SSA. Each model has different computational time steps that are used; in order to take this difference into account, the table shows the time required for one time point per system. For each system, at least 10,000 time steps were used. As observed, the total amount of time that is required for LMEs approach is significantly lower than for SSA.

Among all systems, the LMEs approach requires less computational time to solve the two multistable systems (the Schlögl and the Wilhelm’s models). The LMEs approach is significantly faster than SSA (at least 4000 times faster, Table 2) and advantageous especially for multistable systems. As for oscillatory dynamics, they do not seem to affect the computational time of the LMEs approach.

The number of components, on the other hand, affects significantly the computational time of the LMEs approach. The Wilhelm’s and Brusselator models (both two-component systems) are slower than the Schlögl model (one-component system) and faster that the viral infection model (three-component system).

### 3.5. Dependence on State Space Size

The proposed method is a numerical method to solve stochastic reaction networks. The LMEs include summations across all the possible values of the state space. The state space needs to be specified and be finite in order for the algorithm to perform numerical calculations. This is a known case with numerical methods [22,47] and it can raise concerns about the accuracy of a method, especially in the case of systems with unknown state space. Some ways to calculate the appropriate state space of stochastic networks have been discussed in the literature [11,47].

**Figure 5:**
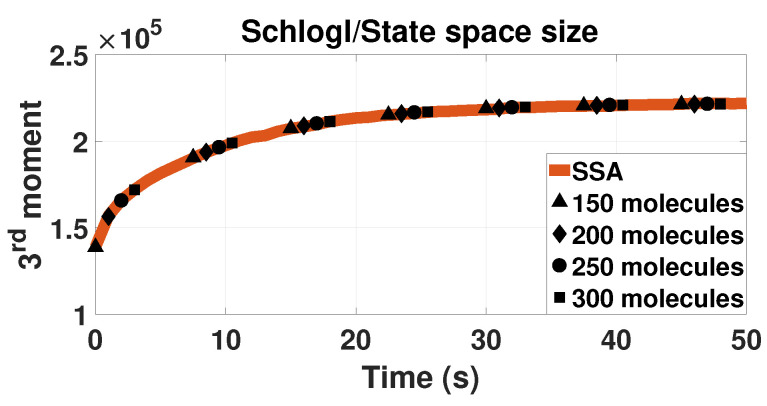
Time evolution of the third-order moment for the Schögl model. The plot is similar to the bottom plot of Figure 1. Here, four different state space lengths are plotted. All the cases have zero as the lower bound of the state space. The coloring in this picture is reverse than the rest of them. The solid orange line is the SSA solution for 500,000 trajectories. The black dots are results with tenth-closure LMEs (M=10). The triangles represent the solution with 150 molecules as the upper bound of the state space, diamonds use 200 molecules limit, circles 250 molecules and the squares 300 molecules.

To evaluate the stability of the Lagrange multipliers approach with the state space size, we use the Schlögl model as an example. The model has one component and thus the connection between state space size and solution accuracy can be drawn easily; yet it has a complex enough bistable behavior. Figure 5 shows results of the LMEs approach for different state space sizes. The solutions are also compared with the SSA results. The accuracy of the method is not affected by the state space size in this example. All different state space lengths provide the same value for the moments of the probability.

## 4. Discussion

Stochasticity governs reaction networks away from the thermodynamic limit. A common approach to solve stochastic chemically reacting systems is through moment equations. However, moment equation systems are not closed for non-linear chemical reactions, and require important assumptions about the form of the probability function.

Here, we present an alternative method to solve stochastic reaction networks by using Lagrange multipliers. An approach to transform moment equations into a closed system of Lagrange multiplier equations (LMEs) is described. Since, LMEs is a closed system, Runge-Kutta methods (e.g., Durmand-Prince RK5(4)7M) can be employed to solve the transient problem and calculate the Lagrange multipliers for different time points. With the knowledge of the Lagrange multipliers, one then can calculate the probability distribution and its moments at any given time.

We show that this is a novel approach to bypass numerical challenges with chemical master equations and moment equations. The only assumption used is that the entropy of the system is maximum at all times. The LMEs solutions are as accurate as alternative methods, such as SSA, with significant computational advantages. Four different non-linear reaction networks are employed to demonstrate the advantages of the new LMEs approach. The networks vary in numbers of components (from one to three) and complexity in behavior (including multistability and oscillations). The number of stable solutions of the network does not affect the accuracy of the LMEs approach. On the other hand, the number of the network’s components can affect the method’s computational requirements. Future studies may be required to assess how the method scales with the size of the reaction network.

For all of the examples studied, the LMEs approach is faster than SSA. Notably, the method is computationally efficient for multistable systems, which are of interest to a large community [42,43,44]. The method also appears unaffected by the size of the system’s state space; the method is stable and accurate for multiple state space sizes. On the other hand, oscillatory behaviors can reduce the method’s accuracy.

## Figures and Tables

**Table 1:** The table shows the reaction networks with their kinetic constants for the bistable Schlögl, multistable Wilhelm, oscillatory Brusselator and viral infection networks. The kinetic constants values and initial condition for each system are also presented. For the third order reactions the kinetic constants are in (molecules${}^{2}$· s)${}^{-1}$ units, for the second order in (molecules · s)${}^{-1}$, for the first order in s${}^{-1}$ and for the zeroth order in molecules · s${}^{-1}$. For the Schlögl model the initial distribution is bimodal. All the other systems have a unimodal initial distribution. The first and second-order factorial moments of the initial distribution is reported for each network.

			Initial Moments	
Models	Reactions	Kinetic Constants	First-Order	Second-Order
**Schlögl**	$3X\overset{k_{1}}{⟶}2X$	$k_{1}=1.5\times 10^{-3}$	{X}=38.01	$\left\{X^{2}\right\}=2.10\times 10^{3}$
	$2X\overset{k_{2}}{⟶}3X$	$k_{2}=1.5\times 10^{-1}$		
	$X\overset{k_{3}}{⟶}\emptyset$	$k_{3}=3.5$		
	$\emptyset \overset{k_{4}}{⟶}X$	$k_{4}=22$		
**Wilhelm**	$Y\overset{k_{1}}{⟶}2X$	$k_{1}=35$	{X}=15.94 {Y}=7.14	$\left\{X^{2}\right\}=2.55\times 10^{2}$ $\left\{XY\right\}=1.24\times 10^{2}$ $\{Y^{2}\}=50.93$
	$2X\overset{k_{2}}{⟶}X+Y$	$k_{2}=1$		
	$X+Y\overset{k_{3}}{⟶}Y$	$k_{3}=1$		
	$X\overset{k_{4}}{⟶}\emptyset$	$k_{4}=9.74$		
	$\emptyset \overset{k_{5}}{⟶}X$	$k_{5}=30$		
**Brusselator**	$\emptyset \overset{k_{1}}{⟶}X$	$k_{1}=10$	{X}=10.68 {Y}=23.40	$\left\{X^{2}\right\}=1.46\times 10^{2}$ $\left\{XY\right\}=2.11\times 10^{2}$ $\left\{Y^{2}\right\}=5.79\times 10^{2}$
	$2X+Y\overset{k_{2}}{⟶}3X$	$k_{2}=9\times 10^{-3}$		
	$X\overset{k_{3}}{⟶}Y$	$k_{3}=3$		
	$X\overset{k_{4}}{⟶}\emptyset$	$k_{4}=1$		
Viral Infection* D=DNA P=Protein R=RNA	$D+P\overset{k_{1}}{⟶}\emptyset$	$k_{1}=1$	{D}=15.41 {P}=25.53 {R}=49.56	$\left\{D^{2}\right\}=2.37\times 10^{2}$ $\left\{DP\right\}=3.89\times 10^{2}$ $\left\{DR\right\}=7.65\times 10^{2}$ $\left\{P^{2}\right\}=6.42\times 10^{2}$ $\left\{PR\right\}=1.26\times 10^{3}$ $\left\{R^{2}\right\}=2.41\times 10^{3}$
	$D\overset{k_{2}}{⟶}R+D$	$k_{2}=3$		
	$R\overset{k_{3}}{⟶}\emptyset$	$k_{3}=1$		
	$R\overset{k_{4}}{⟶}D+R$	$k_{4}=10$		
	$R\overset{k_{5}}{⟶}P+R$	$k_{5}=110$		
	$P\overset{k_{6}}{⟶}\emptyset$	$k_{6}=200$		

**Table 2:** The table presents the results of the LMEs solved with RK5(4)M compared to the ones obtained from SSA alongside the required computational time for each reaction network of Table 1. Each SSA simulation used 500,000 trajectories. This number of trajectories is sufficient for the errors for the mean and variance to be at most O(1)molecules and $O\left(1\right)\;{\mathrm{molecules}}^{2}$, respectively. The first three columns show the average error between the two methods, which is calculated for each time point until steady state is reached and then averaged across all of them. For the probability distribution (P(X)), the Kullback-Leibler divergence [41] is used. For the first and second-order moments the second norm is used. The last three columns present the time that is required for each method. Each system has different initial and final time as well as different step size. To take into account these differences, we report the solving time per each method’s time step in CPU seconds. The last column presents the ratio of SSA time required over the RK5(4)M time.

Network	Average Error (%) of			Time per Step (CPU s)		
	P(X)	1st-Orde Moments	2nd-Order Moments	LMEs	SSA	Time Ratio
**Schlögl**	0.05	0.22	0.22	0.02	419.2	20,960
**Wilhelm**	0.61	0.56	0.71	0.15	732.75	4,885
**Brusselator**	9.90	2.81	6.07	0.74	68.05	92
**Viral Infection**	0.43	0.01	0.02	8.97	64.96	7

## Appendix A. Inverse of Jacobian

The Jacobian matrix *J* of the moment equations for a stochastic reaction network is calculated as:

(A1) $$J_{i,j}=\frac{\partial \mu_{i}}{\partial \lambda_{j}}=-\mu_{i,j}+\mu_{i}\mu_{j}$$

By the definition (Equation (8)), it is true that:

(A2) $$\mu_{i,j}=\sum_{\Omega }\left\{f_{\mu_{i}}f_{\mu_{j}}\left(X\right)\exp\left[-\sum_{j=0}^{M}\lambda_{j}f_{\mu_{j}}\left(X\right)\right]\right\}$$

Thus, $\mu_{i,j}=\mu_{j,i}$. It is then apparent that the Jacobian matrix is symmetric since:

(A3) $$J_{i,j}=-\mu_{i,j}+\mu_{i}\mu_{j}=-\mu_{j,i}+\mu_{j}\mu_{i}=J_{j,i}$$

As a symmetric matrix, J can be calculated as [48]:

(A4) $$J=Q^{T}\Lambda Q$$

where Λ is a diagonal matrix with the diagonal elements being the eigenvalues of *J* and *Q* the associated eigenvector matrix. *Q* is also real orthogonal [48], i.e., $Q^{-1}=Q^{T}$. The inverse of the Jacobian matrix, $J^{-1}$ can be calculated as:

(A5) $$J^{-1}={\left(Q^{T}\Lambda Q\right)}^{-1}={\left(Q\right)}^{-1}{\left(\Lambda \right)}^{-1}{\left(Q^{T}\right)}^{-1}=Q^{T}{\left(\Lambda \right)}^{-1}Q$$

Since Λ is diagonal, ${\left(\Lambda \right)}^{-1}$ is also diagonal with elements the reciprocal elements of Λ (${\left(\Lambda_{i,j}\right)}^{-1}=\frac{1}{\Lambda_{i,j}}$). Additionally, each element of Λ, $\Lambda_{i,j}$, represents each eigenvalue of the Jacobian *J*.

Based on Equation (A5), in order to calculate the inverse of the Jacobian matrix, one only needs to solve the eigenproblem of the Jacobian.

## Appendix B. Runge-Kutta

As mentioned, the LMEs problem is an initial value problem. A proposed way to solve the system is the Dormand-Prince RKF5(4)7M Runge-Kutta method [32]. RKF5(4)7M is an explicit numerical method with adaptive step size. Dormand and Prince formulated the method in order to create a stable approach for local extrapolations; this is the reason it is chosen in this work as it will help to create more stable solution for time calculations by extrapolating the knowledge of past time solutions. A table that includes all the constants of the RKF5(4)7M method (often called “Butcher tableau" [49] or simply “tableau") can be found in the Appendix B.1.

As derived in Equation (15), the LMEs are:

(A6) $$\frac{\partial \lambda }{\partial t}=J{\left(\lambda \right)}^{-1}\left[A\mu (\lambda )+A^{\prime }\mu^{\prime }\left(\lambda \right)+\mu_{c}\right]=F(\lambda )$$

where ***F*** denotes the functional form of the LMEs.

The calculation of Lagrange multipliers, λ, is desired for multiple time points. To calculate the multipliers at the time point n+1, the knowledge of the multipliers at time *n* is required. The Lagrange multipliers at time n+1, $\lambda_{n+1}$, are calculated as:

(A7) $$\lambda_{n+1}=\lambda_{n}+\sum_{i=1}^{7}{\hat{b}}_{i}k_{i}$$

where $\lambda_{n+1}$ are the Lagrange multipliers at time *n*, ${\hat{b}}_{i}$ are constants of RKF5(4)7M and $k_{i}$ are the Runge-Kutta coefficients:

(A8) $$k_{i}=hF\left(\lambda_{n}+\sum_{j=1}^{i-1}a_{ij}k_{j}\right)$$

*h* is the adaptive step size and $a_{ij}$ are RKF5(4)7M constants.

In RKF5(4)7M, a lower-order solution, $\lambda^{*}$, is used to evaluate the error of the solution λ and change accordingly the adaptive step size h. The lower-order solution at time n+1, $\lambda_{n+1}^{*}$, are calculated as:

(A9) $$\lambda_{n+1}^{*}=\lambda_{n}^{*}+\sum_{i=1}^{7}b_{i}k_{i}$$

where $b_{i}$’s are another set of RKF5(4)7M constants. All the constants ($a_{ij}$,${\hat{b}}_{i}$ & $b_{i}$) can be found in the Appendix B.1.

More details about the algorithm of RKF5(4)7M used in this work can be found in the Appendix B.2.

### Appendix B.1. Runge-Kutta Tableau

In this section we list the Runge-Kutta RK5(4)7M tableau. The method was first described by Dormand and Prince [32] and we just list it here for the convenience of the reader. The Runge-Kutta tableau of the method is:

		j= *1*	*2*	*3*	*4*	*5*	*6*		
*i*	$c_{i}$	$a_{ij}$						${\hat{b}}_{i}$	$b_{i}$
*1*	0							$\frac{35}{384}$	$\frac{5179}{57600}$
*2*	$\frac{1}{5}$	$\frac{1}{5}$						0	0
*3*	$\frac{3}{10}$	$\frac{3}{40}$	$\frac{9}{40}$					$\frac{500}{1113}$	$\frac{7571}{16695}$
*4*	$\frac{4}{5}$	$\frac{44}{45}$	−$\frac{56}{15}$	$\frac{32}{9}$				$\frac{125}{192}$	$\frac{393}{640}$
*5*	$\frac{8}{9}$	$\frac{19372}{6561}$	−$\frac{25360}{2187}$	$\frac{64448}{6561}$	−$\frac{212}{729}$			−$\frac{2187}{6784}$	−$\frac{92097}{339200}$
*6*	1	$\frac{9017}{3168}$	−$\frac{355}{33}$	$\frac{46732}{5247}$	$\frac{49}{176}$	−$\frac{5103}{18656}$		$\frac{11}{84}$	$\frac{187}{2100}$
*7*	1	$\frac{35}{384}$	0	$\frac{500}{1113}$	$\frac{125}{192}$	−$\frac{2187}{6784}$	$\frac{11}{84}$	0	$\frac{1}{40}$

### Appendix B.2. Runge-Kutta Algorithm

The proposed algorithm to solve LMEs based on the Dormand-Prince RKF5(4)M method is:

1. Calculate vector $\mu_{c}$ and matrices *A* and $A^{\prime }$ as in [17]. These matrices depend only on the kinetic constants of the system and they are constant for all time points.
2. Introduce the time step size *h*. The time point of this iteration is $t_{n+1}=t_{n}+h$, where $t_{n}$ is the time point of the previous iteration.
3. Increase the iteration counter by 1. For the rest of the algorithm the iteration step will be denoted by n+1. At the first iteration, n=0.
4. Introduce the previous step (iteration *n*) Lagrange multipliers $\lambda_{n}$. At the first iteration, introduce the initial condition $\lambda_{0}$. The subscripts here refers to the time step and are applied on the whole Lagrange multiplier vector.
5. Calculate the $k_{i}$’s coefficients from equation $k_{i}=hF\left(\lambda_{n}+\sum_{j=1}^{i-1}a_{ij}k_{j}\right)$ and the tableau (Appendix B.1). Each coefficient $k_{i}$ depends on the functional form ***F*** of the Lagrange multiplier equations ($F(\lambda )=J{\left(\lambda \right)}^{-1}\left[A\mu (\lambda )+A^{\prime }\mu^{\prime }\left(\lambda \right)+\mu_{c}\right]$) and on different set of Lagrange multipliers ($\lambda_{n}+\sum_{j=1}^{i-1}a_{ij}k_{j}$). To account for the change of the Lagrange multipliers, there is a need for a subroutine to calculate $k_{i}$.
6. This is a proposed subroutine for $k_{i}$
  (a) Calculate the augmented Lagrange multipliers for the specific $k_{i}$, $\lambda_{n,k_{i}}=\lambda_{n}+\sum_{j=1}^{i-1}a_{ij}k_{j}$. The values of $a_{ij}$ can be found in the tableau in Appendix B.1.
  (b) Calculate the lower ($\mu (\lambda_{n,k_{i}})$) and higher-order moments ($\mu^{\prime }\left(\lambda_{n,k_{i}}\right)$) for the augmented Lagrange multipliers based on equation $\mu_{i}=\sum_{\Omega }f_{\mu_{i}}\left(X\right)\exp\left(-1-\sum_{j}\lambda_{j}f_{\mu_{j}}\left(X\right)\right)$.
  (c) Calculate the inverse of the Jacobian, $J{\left(\lambda_{n,k_{i}}\right)}^{-1}$ for the augmented multipliers as described in Appendix A.
  (d) Calculate the functional form $F(\lambda_{n,k_{i}})$ of the Lagrange multiplier equations ($F(\lambda )=J{\left(\lambda \right)}^{-1}\left[A\mu (\lambda )+A^{\prime }\mu^{\prime }\left(\lambda \right)+\mu_{c}\right]$).
  (e) Calculate $k_{i}$ based on equations $k_{i}=hF(\lambda_{n,k_{i}})$.
7. Repeat step 6 for all seven $k_{i}$ coefficients, i=1,…,7. Its coefficient $k_{i}$ depends on all the previous coefficients, thus the calculation of each one needs to be in sequence and include the subroutine. For example, the calculations of step 6 should first performed for i=1, then based on $k_{1}$ to be performed for i=2, then based on $k_{2}$ to be performed for i=3 etc.
8. Calculate the Lagrange multipliers for the current iteration n+1, $\lambda_{n+1}$, based on equation $\lambda_{n+1}=\lambda_{n}+\sum_{i=1}^{7}{\hat{b}}_{i}k_{i}$ and the tableau (Appendix B.1).
9. Calculate the lower-order solution for the current iteration n+1, $\lambda_{n+1}^{*}$, based on equation $\lambda_{n+1}^{*}=\lambda_{n}+\sum_{i=1}^{7}b_{i}k_{i}$ and the tableau (Appendix B.1).
10. Check the accuracy of the solution by calculating the error ϵ:
  (A10) $$\epsilon ={\left|\frac{\lambda_{n+1}-\lambda_{n+1}^{*}}{\lambda_{n+1}}\right|}_{2}$$
11. If the error ϵ is smaller than the wanted tolerance accept the solution and proceed to the next step. Otherwise, set a new time step size h=h·s and go back to step 5 and re-perform the calculation with the new step size. *s* is used to adjust the step size based on the accuracy of the solution and is given by:
  (A11) $$s={\left(\frac{tolerance\cdot h}{2\cdot |\lambda_{n+1}^{*}-\lambda_{n+1}{|}_{2}}\right)}^{\frac{1}{4}}$$
12. Accept the solution of Lagrange multipliers for the current iteration (n+1) and current time point ($t_{n+1}$) as calculated at step 8.
13. Set the new step size to h=h·s and proceed to step 3 to calculate the solution for the rest of the time points until the final is reached.
14. For any given time *t*, the probability distribution and its moments can be calculated based on the Lagrange multipliers (λ(t)) and equations $P\left(X\right)=\exp\left[-1-\sum_{j}\lambda_{j}f_{\mu_{j}}\left(X\right)\right]$ & $\mu_{i}=\sum_{\Omega }f_{\mu_{i}}\left(X\right)\exp\left(-1-\sum_{j}\lambda_{j}f_{\mu_{j}}\left(X\right)\right)$.
